# Association between KIF1B rs17401966 genetic polymorphism and hepatocellular carcinoma susceptibility: an updated meta-analysis

**DOI:** 10.1186/s12881-019-0778-y

**Published:** 2019-04-04

**Authors:** Ying-ying Luo, Hong-peng Zhang, Ai-long Huang, Jie-li Hu

**Affiliations:** 1 Intensive Care Unit, The Third People’s Hospital of Chengdu, The Second Affiliated Hospital of Chengdu, Chongqing Medical University, Chengdu, Sichuan China; 2 Institute for Viral Hepatitis, Key Laboratory of Molecular Biology on Infectious Diseases, Ministry of Education, The Second Affiliated Hospital of Chongqing Medical University, Chongqing, China

**Keywords:** KIF1B, Polymorphism, Hepatocellular carcinoma, Liver cancer

## Abstract

**Background:**

Several studies have focused on the association between KIF1B rs17401966 polymorphism and susceptibility to hepatitis B virus-related (HBV-related) hepatocellular carcinoma (HCC), but the conclusions have been inconsistent. We have conducted this updated meta-analysis to explore the association between KIF1B rs17401966 polymorphism and HCC susceptibility.

**Methods:**

Eligible studies were identified through systematic searches in PubMed, OVID, ISI Web of Science, Chinese National Knowledge Infrastructure, and Wanfang databases. The quality of evidence was systematically assessed by use of the Newcastle-Ottawa Scale for case control studies in meta-analyses.

**Results:**

Ten studies containing 18 independent case-control studies were included. The results revealed a significant association between KIF1B rs17401966 polymorphism and susceptibility to HCC under a random-effect allelic model (OR = 0.85, 95% CI 0.76–0.94, *P* = 0.003); HBV-positive subgroup (OR = 0.82, 95% CI 0.72–0.95, *P* = 0.007); and Chinese-subgroup (OR = 0.82, 95% CI 0.72–0.93, *P* = 0.002).

**Conclusions:**

G-allele appears to be a protective allele of KIF1B for HCC, especially in HBV-positive and Chinese populations. More well-designed studies with larger sample size and various ethnic groups and risk factors are needed to establish that KIF1B rs17401966 polymorphism is significantly associated with risk of HCC.

**Electronic supplementary material:**

The online version of this article (10.1186/s12881-019-0778-y) contains supplementary material, which is available to authorized users.

## Background

Hepatocellular carcinoma (HCC) is a leading cause of cancer-related death, with an estimated 700,000 deaths and 750,000 new cases worldwide per year, and these numbers are expected to increase with time [1]. Moreover, the prognosis of HCC is very unfavorable, with the five-year survival rate less than 10% [2].

HCC is a complex process, associated with many factors and co-factors, including genetic predisposition, environmental factors, and viruses, among which hepatitis B virus (HBV) contributes the biggest [2, 3]. Among these factors, increases in allelic losses, chromosomal changes and gene mutations appear to be crucial molecular and pathogenic steps in the development of HCC. Kinesin Family Member 1B (KIF1B) is a tumor suppressor in many cancers, including those of liver, colon, breast, and brain (aggressive neuroblastoma), and pheochromocytoma [4, 5]. KIF1B is suspected of playing a role also in the development and progress of HCC: Several reports have focused on the association between KIF1B rs17401966 polymorphism and susceptibility to HCC; however, conclusions of the studies are inconsistent. There were two meta-analyses on the associations between KIF1B rs17401966 polymorphism and HCC [6, 7], and a meta-analysis on the associations between KIF1B rs17401966 polymorphism and HBV-related HCC [8], were published in the last 2 years. As new papers published in the last 5 years, we have performed a updated meta-analysis to assess the relationship of KIF1B rs17401966 polymorphism and HCC.

## Methods

### Search strategy

Eligible studies were identified systematically from PubMed, OVID, ISI Web of Science, Chinese National Knowledge Infrastructure, and Wanfang databases up to June 21, 2016, written in English or Chinese. The search terms used were: KIF1B, rs17401966, liver cancer, hepatocellular carcinoma, and polymorphism. Two researchers independently investigated the titles, abstracts and full texts of relevant studies. The results were compared, and disagreements were resolved by consensus.

### Inclusion and exclusion criteria

The inclusion criteria were: a) case-control studies; b) articles that evaluated the association between KIF1B rs17401966 polymorphism and risk of HCC; c) articles that provided sufficient data to estimate an odds ratio (OR) and corresponding 95% confidence interval (CI); d) English or Chinese language; e) solid evidence for HCC; and f) HBV as an HCC subgroup. Unpublished reports, abstracts, reviews, meta-analyses, letters, case reports and animal studies were excluded. When studies had overlap or included the same subjects the latest or the most complete study was selected.

### Characteristics of included studies

Ten records were identified through database searches. Finally, 10 studies [9–18], containing 18 independent case-control studies, based on the inclusion and exclusion criteria, were included.

In total, 18,893 participants were selected (8427 HCC cases and 10,466 controls). Characteristics of the included studies in the meta-analysis, including ethnicity, language, number of cases and controls, source of controls, matching factors, genotyping method, and Hardy-Weinberg Equilibrium of the included cohorts are shown in Table 1.

**Table 1:** Characteristics of the included studies in the meta-analysis

Study	Ethnicity	Language	Cases/controls	HBV-positive cases/controls	Source of controls	Matching factors	Genotyping method	HWE^a^
Zhang 2010 Guangxi	Chinese	English	348/359	348/359	Population based	Age, sex, geographic regions	Affymetrix Genome-Wide Human SNP Array5.0	Yes
Zhang 2010 Beijing	Chinese	English	276/266	276/266	Population based	Age, sex, geographic regions	SNPstream 12-plex Genotyping System	Yes
Zhang 2010 Jiangsu	Chinese	English	507/215	507/215	Population based	Age, sex, geographic regions	TaqMan	Yes
Zhang 2010 Guangdong	Chinese	English	751/509	751/509	Hospital based	Age, sex, geographic regions	TaqMan	Yes
Zhang 2010 Shanghai	Chinese	English	428/440	428/440	Hospital based	Age, sex, geographic regions	TaqMan	Yes
Hu 2012	Chinese	English	1293/2671	1293/1334	Population based	Age, sex	TaqMan	Yes
Li 2012 Central	Chinese	English	480/484	480/484	Population based	Age, sex, geographic regions	iPLEX, TaqMan	Yes
Li 2012 Southern	Chinese	English	1058/981	1058/981	Population based	Age, sex, geographic regions	iPLEX, TaqMan	Yes
Sawai 2012 Japan1	Japanese	English	179/769	179/769	Population based	–	PCR-based Invader assay	Yes
Sawai 2012 Japan2	Japanese	English	142/251	142/251	Hospital based	–	TaqMan	Yes
Sawai 2012 Korea	Korean	English	164/144	164/144	Population based	–	TaqMan	Yes
Sawai 2012 Hong Kong	Chinese	English	93/187	93/187	Hospital based	–	TaqMan	Yes
Chen 2013	Chinese	English	503/772	503/772	Hospital based	Age, sex	TaqMan	Yes
Jiang 2013	Chinese	English	1161/1353	1161/1353	Population based	–	MassARRAY, TaqMan	Yes
Sopipong 2013	Thais	English	202/196	202/196	Hospital based	–	TaqMan	Yes
Su 2014	Chinese	English	160/160	0/0	Population based	Age, sex	iPLEX	Yes
Pan 2015	Chinese	Chinese	376/403	101/11	Hospital based	Age, sex, geographic regions	MassARRAY	Yes
Chen 2016	Chinese	English	306/306	229/54	Hospital based	Age, sex	TaqMan	Yes

^a^HWE, Hardy-Weinberg Equilibrium

Quality assessment of case-control studies included in the meta-analysis was determined with NOS. As shown in Table 2, quality scores ranged from 6 to 9, indicating that all included studies had high-quality scores.

**Table 2:** The Newcastle-Ottawa Scale for assessing the quality of case-control studies

Study included	Case defined adequately	Represent-ativeness of the cases	Community controls	Controls have no history of the outcome	Study controls for age	Study controls for sex	Ascertainment of exposure with secure record	Ascertainment of exposure with structured interview where blind to case/control status	Same method of ascertainment for cases and controls	Same non-response rate for both groups	Total score
Zhang 2010 Guangxi	1	1	1	1	1	1	1	0	1	1	9
Zhang 2010 Beijing	1	1	1	1	1	1	1	0	1	0	8
Zhang 2010 Jiangsu	1	1	1	1	1	1	1	0	1	0	8
Zhang 2010 Guangdong	1	1	0	1	1	1	1	0	1	1	8
Zhang 2010 Shanghai	1	1	0	1	1	1	1	0	1	0	7
Hu 2012	1	1	1	1	1	1	1	0	1	1	9
Li 2012 Central	1	1	1	1	1	1	0	0	1	1	8
Li 2012 Southern	1	1	1	1	1	1	0	0	1	1	8
Sawai 2012 Japan1	1	1	1	1	0	0	1	0	1	1	7
Sawai 2012 Japan2	1	1	0	1	0	0	1	0	1	1	6
Sawai 2012 Korea	1	1	1	1	0	0	1	0	1	1	7
Sawai 2012 Hong Kong	1	1	0	1	0	0	1	0	1	1	6
Chen 2013	1	1	1	1	1	1	1	0	1	1	9
Jiang 2013	1	1	1	1	0	0	1	0	1	1	7
Sopipong 2013	1	1	1	1	0	0	1	0	1	0	6
Su 2014	1	1	1	1	1	1	1	0	1	1	9
Pan 2015	1	1	0	1	1	1	1	0	1	0	7
Chen 2016	1	1	0	1	1	1	1	0	1	0	7

### Data extraction

Two researchers independently extracted these data: the first author’s surname; year of publication; country of region; ethnicity; language; total number of cases and controls; source of controls; matching factors; and genotype method. Study quality was assessed with use of the Newcastle-Ottawa Scale (NOS) [19]. The NOS assessment for case control studies was appropriate; a study was regarded as a high-quality study when it rated six or more stars.

### Statistical methods

The Hardy-Weinberg Equilibrium was calculated for control groups of each study, using the goodness-of-fit *χ*^2^ -test. *P* < 0.05 was considered deviation from Hardy-Weinberg Equilibrium. Meta-analyses were conducted with Stata 14.0 (StataCorp, College Station, TX, USA). The strength of the association between KIF1B rs17401966 polymorphism and HCC susceptibility was measured by OR and corresponding 95% CI. Traditionally, meta-analysis on genetic association studies were based on nearly all genetic models, which not only increase the probability of false-positive rate but also making the explanation of results more confused. According to the Ammarin Thakkinstian’s theory, that is to say if OR1 < OR2 < 1 and OR1 < OR3 < 1, then a co-dominant model is suggested [20], we determined co-dominant model is the best genetic model. The pooled OR and 95% CI were calculated under the allelic model (G-allele vs A-allele) and co-dominant genotype model (GG vs AA, AG vs AA, GG vs AG). The statistical significance of the pooled OR was determined by the Z test; *P* < 0.05 was considered statistically significant. Heterogeneity was assessed by use of the *I*^2^ statistic. When *I*^2^ was > 50%, the heterogeneity was considered statistically significant and a random-effect model was applied to the meta-analysis; otherwise, a fixed-effect model was used. The risk of publication bias was determined with the Begg’s rank correlation test and Egger’s linear regression test (*P* < 0.05 was considered statistically significant in both) by Stata 14.0. All *p* values were measured from two-tailed tests of statistical significance with a type I error rate of 5%.

Artwork was created with CorelDRAW X7 (Corel Corporation, Ottawa, Canada).

## Results

**Fig. 1:**
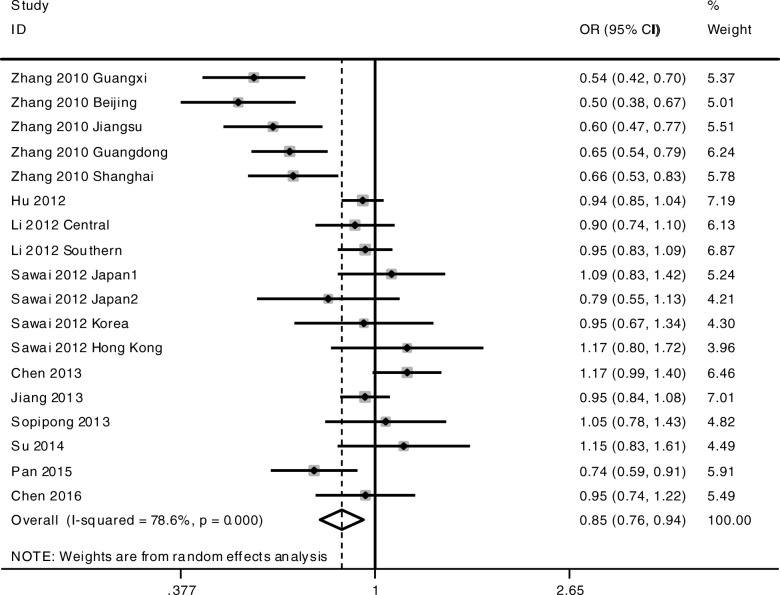
Forest plots of association between KIF1B polymorphism and HCC susceptibility. Forest plots were conducted under the allelic model G-allele vs A-allele

**Fig. 2:**
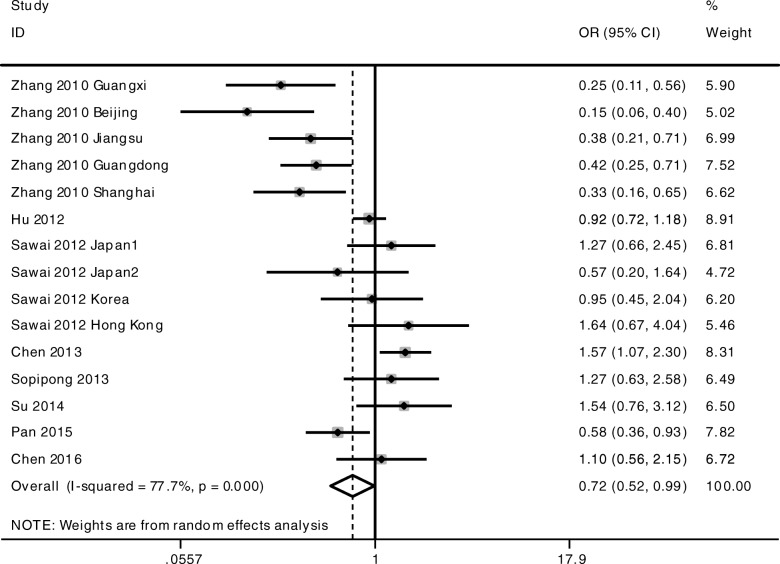
Forest plots of association between KIF1B polymorphism and HCC susceptibility. Forest plots were conducted under the co-dominant genotype model GG vs AA

**Fig. 3:**
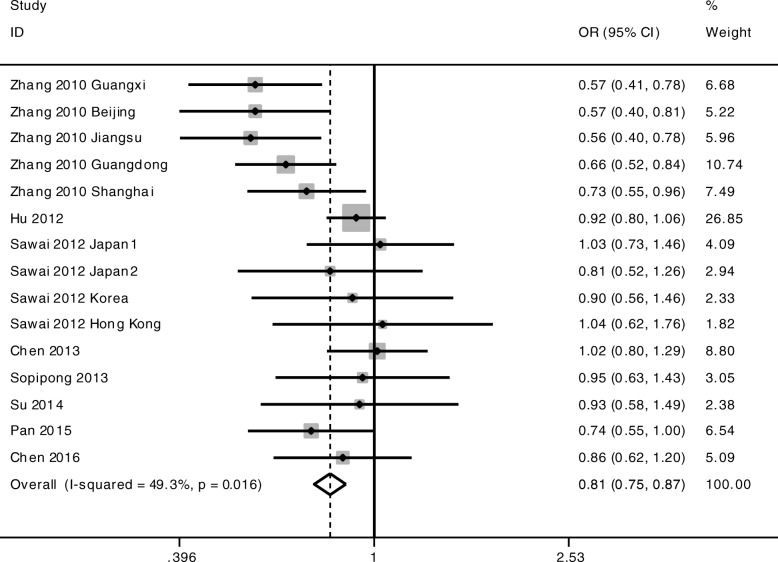
Forest plots of association between KIF1B polymorphism and HCC susceptibility. Forest plots were conducted under the co-dominant genotype model AG vs AA

The meta-analysis was conducted among all the cohorts under the allelic model (G-allele vs A-allele) and co-dominant models (GG vs AA, GG vs AG, AG vs AA). The statistical significance of the pooled OR was determined by the Z test; *P* < 0.05 was considered statistically significant. All *p* values were measured from two-tailed tests of statistical significance with a type I error rate of 5%. As shown in Fig. 1 and Table 3, a significant allelic association was recorded under a random-effect allelic model, with OR = 0.85 (95% CI 0.76–0.94, *P* = 0.003), indicating that the G-allele is a protective allele of KIF1B for HCC compared to A-allele. Similar results were found under the co-dominant genotype models GG vs AA (OR = 0.72, 95% CI 0.52–0.99, *P* = 0.044) (Fig. 2, Table 3) and AG vs AA (OR = 0.81, 95% CI 0.75–0.87, *P* < 0.001) (Fig. 3, Table 3). No statistical differences were found under the co-dominant genotype model GG vs AG (Table 3).

**Table 3:** Overall meta-analysis results with subgroup conducted by HBV status and ethnicity

Outcome/subgroup	Case	Control	Case vs Control			Heterogeneity		Egger’s test	Begg’s test
			OR	95% CI	P	I^2^	P	P	P
G-allele vs A-allele									
All	18,710	23,204	0.85	0.76–0.94	0.003	78.6%	< 0.001	0.307	0.649
HBV positive	12,584	13,852	0.82	0.72–0.95	0.007	81.9%	0.000		
HBV negative	4270	7080	0.91	0.79–1.06	0.236	50.5%	0.109		
Chinese	15,480	18,212	0.82	0.72–0.93	0.002	82.6%	< 0.001		
Non-Chinese	1374	2720	0.99	0.84–1.15	0.855	0.0%	0.531		
GG vs AA									
All	4241	5301	0.72	0.52–0.99	0.044	77.7%	< 0.001	0.249	0.488
HBV positive	2377	2514	0.63	0.39–1.01	0.057	81.8%	0.000		
HBV negative	1331	2131	0.91	0.65–1.29	0.603	50.0%	0.112		
Chinese	3268	3775	0.64	0.43–0.95	0.028	82.9%	< 0.001		
Non-Chinese	440	870	1.07	0.73–1.56	0.729	0.0%	0.538		
AG vs AA									
All	6185	8085	0.81	0.75–0.87	< 0.001	49.3%	0.016	0.686	0.843
HBV positive	3387	3802	0.76	0.66–0.89	0.001	54.9%	0.014		
HBV negative	1949	3222	0.88	0.79–0.99	0.036	0.0%	0.644		
Chinese	4705	5754	0.76	0.66–0.87	< 0.001	59.7%	0.006		
Non-Chinese	631	1270	0.93	0.76–1.15	0.518	0.0%	0.868		
GG vs AG									
All	2886	4182	0.91	0.72–1.15	0.422	53.3%	0.008	0.253	0.692

**Fig. 4:**
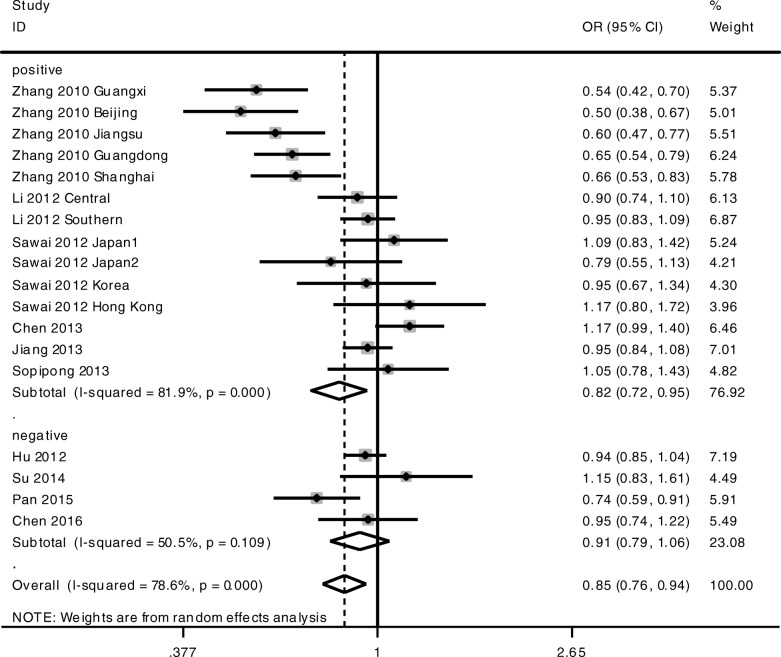
Forest plots of association between KIF1B polymorphism and HCC susceptibility. Forest plots were conducted under the allelic model G-allele vs A-allele stratified by HBV status

**Fig. 5:**
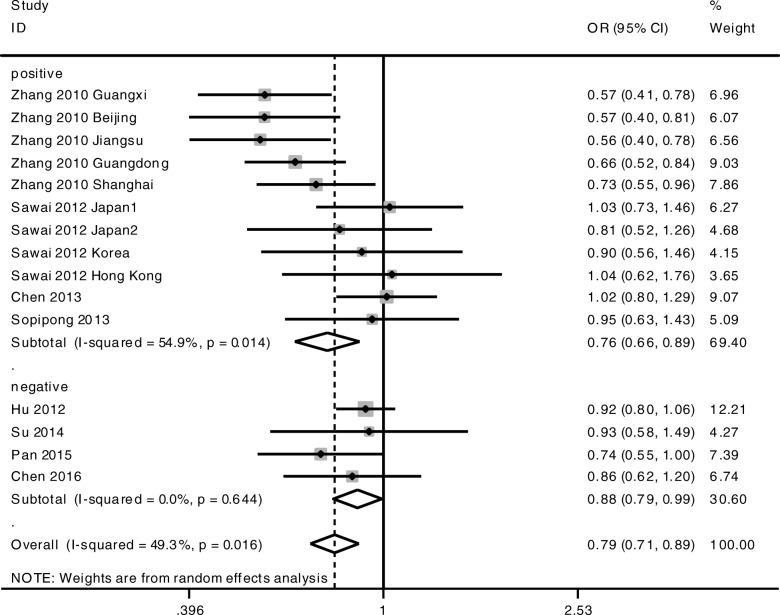
Forest plots of association between KIF1B polymorphism and HCC susceptibility. Forest plots were conducted under the co-dominant genotype model AG vs AA stratified by HBV status

As shown in Table 3, heterogeneity was high, so we performed stratification analysis by HBV status. In the HBV-positive subgroup, KIF1B rs17401966 was associated with HBV-related HCC under allelic model G-allele vs A-allele (OR = 0.82, 95% CI 0.72–0.95, *P* = 0.007) (Table 3, Fig. 4) and co-dominant genotype models AG vs AA (OR = 0.76, 95% CI 0.66–0.89, *P* = 0.001) (Table 3, Fig. 5). No statistical differences were found under GG vs AG or GG vs AG genotype models (Table 3). In the HBV-negative subgroup, KIF1B rs17401966 was associated with HBV-related HCC under co-dominant genotype models AG vs AA (OR = 0.88, 95% CI 0.79–0.99, *P* = 0.036) (Table 3, Fig. 5). No statistical differences were found under other genotype models or allelic model (Table 3).

We further performed a meta-analysis stratified by ethnicity (Table 3): In the Chinese sub-group, KIF1B rs17401966 was associated with HCC under allelic model G-allele vs A-allele (OR = 0.82, 95% CI 0.72–0.93, *P* = 0.002) and co-dominant genotype models GG vs AA (OR = 0.64, 95% CI 0.43–0.95, *P* = 0.028) and AG vs AA (OR = 0.76, 95% CI 0.66–0.87, *P* < 0.001). No statistical differences were found under any models in the non-Chinese subgroup.

Sensitivity analysis was performed by removing the studies in the meta-analysis to evaluate the effects of individual case-control study on the meta-analysis results by Stata 14.0 (Additional files 1, 2, and 3: Figures S1–S3, Additional files 4, 5, and 6: Table S1–S3). The corresponding pooled OR were not changed when any single study was removed, indicating that the statistical results did not suggest significant effects, revealing the stability and credibility of the results.

## Discussion

This meta-analysis was performed to assess the relationship of KIF1B rs17401966 polymorphism and HCC susceptibility. The results revealed a significant association between KIF1B rs17401966 polymorphism and HCC susceptibility under a random-effect allelic model, the HBV-positive subgroup, and Chinese-subgroup. Mutant G-allele and heterozygous mutant genotype AG of KIF1B may be protective against HCC, especially in HBV-positive and Chinese populations.

Although heterogeneity among the studies was high, associations were discovered in a random-effect model as well. High heterogeneity may be due to different gender, ages or duration of infection among populations included in the various studies. Results of the Begg’s rank correlation test and Egger’s linear regression test documented there was no obvious publication bias in the meta-analysis. High quality of the included studies confirms the stability and reliability of our results.

Although two meta-analyses on the associations between KIF1B rs17401966 polymorphism and hepatocellular carcinoma [6, 7] and one meta-analyses on the associations between KIF1B rs17401966 polymorphism and HBV-related hepatocellular carcinoma have been reported in the last 5 years [8]. In 2013, Wang et al. performed a meta-analysis, with a total of 5 studies containing 13 cohorts with 5773 cases and 6404 controls, under the allele model (G vs. A), the co-dominant models (GG vs. AA; GG vs. AG and AG vs. AA), the dominant model (GG + AG vs. AA), and recessive model (GG vs. AG + AA), which suggests the presence of the G allele at rs17401966 of the KIF1B gene may decrease the risk for HCC [6]. In 2014, Zhang et al. performed a meta-analysis, with a total of 15 case-control studies with 7596 HCC cases and 9614 controls. And a significant association between KIF1B rs17401966 and HCC risk was detected (OR = 0.81, 95% CI 0.72–0.91, *P* < 0.001) [7]. In 2017, Su et al. conducted a meta-analysis, with a total of 5 studies containing 12 cohorts with 4886 HCC cases and 5442 controls. Su et al. verify a weak association between the KIF1B rs1740199 polymorphism and HCC risk [8], which is the same with HBV-positive subgroup of our meta-analysis. We conducted the present analysis because the conclusions of those studies were controversial (because of different criteria for inclusion of data, different original studies, different stratified facators and articles written in English only). Moreover, recently published articles on this association needed to be included for reevaluation. Thus, we feel our meta-analysis is up to date and valid.

More than half of all HCCs in the world are secondary to chronic HBV infection [21–24]. A study of 22,707 Chinese men in Taiwan found that the incidence of HCC among carriers of hepatitis B surface antigen is much higher than among non-carriers [25]. Thus, we stratified our meta-analysis by HBV status. As with all patients combined, mutant G-allele and heterozygous mutant genotype AG of KIF1B were potential protective factors for HCC in the HBV-positive subgroup. This conclusion is partly consistent with the meta-analysis conducted by Wang et al. [6]; in HBV-negative subjects, only heterozygous mutant genotype AG was associated with decreased risk of HCC. Although high heterogeneity was present among pooled studies, the association existed also under a random-effect model.

We are aware of some limitations in our meta-analysis. First, not all of the studies reported environmental factors and possible virus co-infection. HCC development is driven by environmental factors, such as alcohol and aflatoxin B1, genetic factors, and viral infections besides HBV infection, such as HCV infection. Not all the included studies assessed these confounding factors [3], so we could not determine their role in HCC development by stratification analysis; more well-designed case-control studies may be needed. Second, not all of the included studies adjusted for potential cofounders, except for ethnicity, such as age and gender. Thus, caution is needed when applying our conclusions to populations of different age, gender and other potential confounding factors.

## Conclusions

In conclusion, the results of this meta-analysis indicated that KIF1B rs17401966 polymorphism is associated with a decreased risk of HCC, especially in HBV-positive and Chinese populations. In order to convincingly establish that KIF1B rs17401966 polymorphism is significantly associated with risk of HCC, future studies should be well-designed, multicenter, with large sample size and a broad range of ethnic groups and risk factors.

## Additional files

Additional file 1: **Figure S1.** Sensitivity analysis of association between KIF1B polymorphism and HCC susceptibility under the allelic model G-allele vs A-allele: The corresponding pooled OR were not changed when any single study was removed. (DOCX 242 kb)

Additional file 2: **Figure S2.** Sensitivity analysis of association between KIF1B polymorphism and HCC susceptibility under the co-dominant genotype model GG vs AA: The corresponding pooled OR were not changed when any single study was removed. (DOCX 220 kb)

Additional file 3: **Figure S3.** Sensitivity analysis of association between KIF1B polymorphism and HCC susceptibility under the co-dominant genotype model AG vs AA: The corresponding pooled OR were not changed when any single study was removed. (DOCX 220 kb)

Additional file 4: **Table S1.** Sensitivity analysis of association between KIF1B polymorphism and HCC susceptibility under the allelic model G-allele vs A-allele: The corresponding pooled OR were not changed when any single study was removed. (DOCX 88 kb)

Additional file 5: **Table S2.** Sensitivity analysis of association between KIF1B polymorphism and HCC susceptibility under the co-dominant genotype model GG vs AA: The corresponding pooled OR were not changed when any single study was removed. (DOCX 81 kb)

Additional file 6: **Table S3.** Sensitivity analysis of association between KIF1B polymorphism and HCC susceptibility under the co-dominant genotype model AG vs AA: The corresponding pooled OR were not changed when any single study was removed. (DOCX 81 kb)

## Abbreviations

CI: Confidence interval
HBV: Hepatitis B virus
HCC: Hepatocellular carcinoma
KIF1B: Kinesin Family Member 1B
NOS: The Newcastle-Ottawa Scale
OR: Odds ratio

## References

[1] Jemal A, Bray F, Center MM, Ferlay J, Ward E, Forman D (2011). Global cancer statistics. CA Cancer J Clin.

[2] McGlynn KA, London WT (2011). The global epidemiology of hepatocellular carcinoma: present and future. Clin Liver Dis.

[3] Levrero M, Zucman-Rossi J (2016). Mechanisms of HBV-induced hepatocellular carcinoma. J Hepatol.

[4] Munirajan AK, Ando K, Mukai A, Takahashi M, Suenaga Y, Ohira M (2008). KIF1Bbeta functions as a haploinsufficient tumor suppressor gene mapped to chromosome 1p36.2 by inducing apoptotic cell death. J Biol Chem.

[5] Schlisio S, Kenchappa RS, Vredeveld LC, George RE, Stewart R, Greulich H (2008). The kinesin KIF1Bbeta acts downstream from EglN3 to induce apoptosis and is a potential 1p36 tumor suppressor. Genes Dev.

[6] Wang ZC, Gao Q, Shi JY, Yang LX, Zhou J, Wang XY (2013). Genetic polymorphism of the kinesin-like protein KIF1B gene and the risk of hepatocellular carcinoma. PLoS One.

[7] Zhang Z (2014). Association between KIF1B rs17401966 polymorphism and hepatocellular carcinoma risk: a meta-analysis involving 17,210 subjects. Tumour Biol.

[8] Su M, Guo J, Huang J (2017). Meta-analysis of the correlation between the rs17401966 polymorphism in kinesin family member 1B and susceptibility to hepatitis B virus related hepatocellular carcinoma. Clin Mol Hepatol.

[9] Zhang H, Zhai Y, Hu Z, Wu C, Qian J, Jia W (2010). Genome-wide association study identifies 1p36.22 as a new susceptibility locus for hepatocellular carcinoma in chronic hepatitis B virus carriers. Nat Genet.

[10] Hu L, Zhai X, Liu J, Chu M, Pan S, Jiang J (2012). Genetic variants in human leukocyte antigen/DP-DQ influence both hepatitis B virus clearance and hepatocellular carcinoma development. Hepatology..

[11] Li S, Qian J, Yang Y, Zhao W, Dai J, Bei JX (2012). GWAS identifies novel susceptibility loci on 6p21.32 and 21q21.3 for hepatocellular carcinoma in chronic hepatitis B virus carriers. PLoS Genet.

[12] Sawai H, Nishida N, Mbarek H, Matsuda K, Mawatari Y, Yamaoka M (2012). No association for Chinese HBV-related hepatocellular carcinoma susceptibility SNP in other East Asian populations. BMC Med Genet.

[13] Chen K, Shi W, Xin Z, Wang H, Zhu X, Wu X (2013). Replication of genome wide association studies on hepatocellular carcinoma susceptibility loci in a Chinese population. PLoS One.

[14] Jiang DK, Sun J, Cao G, Liu Y, Lin D, Gao YZ (2013). Genetic variants in STAT4 and HLA-DQ genes confer risk of hepatitis B virus-related hepatocellular carcinoma. Nat Genet.

[15] Sopipong W, Tangkijvanich P, Payungporn S, Posuwan N, Poovorawan Y (2013). The KIF1B (rs17401966) single nucleotide polymorphism is not associated with the development of HBV-related hepatocellular carcinoma in Thai patients. Asian Pac J Cancer Prev.

[16] Su C, Lin Y, Niu J, Cai L (2014). Association between polymorphisms in tumor suppressor genes and oncogenes and risk of hepatocellular carcinoma: a case-control study in an HCC epidemic area within the Han Chinese population. Med Oncol.

[17] Pan H, Su C, Lin Y, Niu J (2015). The relationship between the KIF1B (rs17401966) single nucleotide polymorphism and the genetic susceptibility to hepatocellular carcinoma. Zhonghua Yu Fang Yi Xue Za Zhi.

[18] Chen JH, Wang YY, Lv WB, Gan Y, Chang W, Tian NN (2016). Effects of interactions between environmental factors and KIF1B genetic variants on the risk of hepatocellular carcinoma in a Chinese cohort. World J Gastroenterol.

[19] GA Wells, B Shea, D O'Connell, J Peterson, V Welch, M Losos, et al. The Newcastle-Ottawa Scale (NOS) for assessing the quality of nonrandomised studies in meta-analyses. Available at: http://www.ohri.ca/programs/clinical_epidemiology/oxford.asp

[20] Thakkinstian A, McElduff P, D'Este C, Duffy D, Attia J (2005). A method for meta-analysis of molecular association studies. Stat Med.

[21] Yang JD, Roberts LR (2010). Hepatocellular carcinoma: A global view. Nat Rev Gastroenterol Hepatol.

[22] Ming L, Thorgeirsson SS, Gail MH, Lu P, Harris CC, Wang N (2002). Dominant role of hepatitis B virus and cofactor role of aflatoxin in hepatocarcinogenesis in Qidong, China. Hepatology..

[23] Perz JF, Armstrong GL, Farrington LA, Hutin YJ, Bell BP (2006). The contributions of hepatitis B virus and hepatitis C virus infections to cirrhosis and primary liver cancer worldwide. J Hepatol.

[24] Raza SA, Clifford GM, Franceschi S (2007). Worldwide variation in the relative importance of hepatitis B and hepatitis C viruses in hepatocellular carcinoma: a systematic review. Br J Cancer.

[25] Beasley RP, Hwang LY, Lin CC, Chien CS (1981). Hepatocellular carcinoma and hepatitis B virus. A prospective study of 22 707 men in Taiwan. Lancet..

