# Supplementary material for: Association between KIF1B rs17401966 genetic polymorphism and hepatocellular carcinoma susceptibility: an updated meta-analysis
Source: BMC Med Genet. 2019 Apr 4;20:59. doi: 10.1186/s12881-019-0778-y (PMC6449895; doi:10.1186/s12881-019-0778-y)
Supplement: Supplementary file 1 — Figure S1. Sensitivity analysis of association between KIF1B polymorphism and HCC susceptibility under the allelic model G-allele vs A-allele: The corresponding pooled OR were not changed when any single study was removed. (DOCX 242 kb) [file 12881_2019_778_MOESM1_ESM.docx]

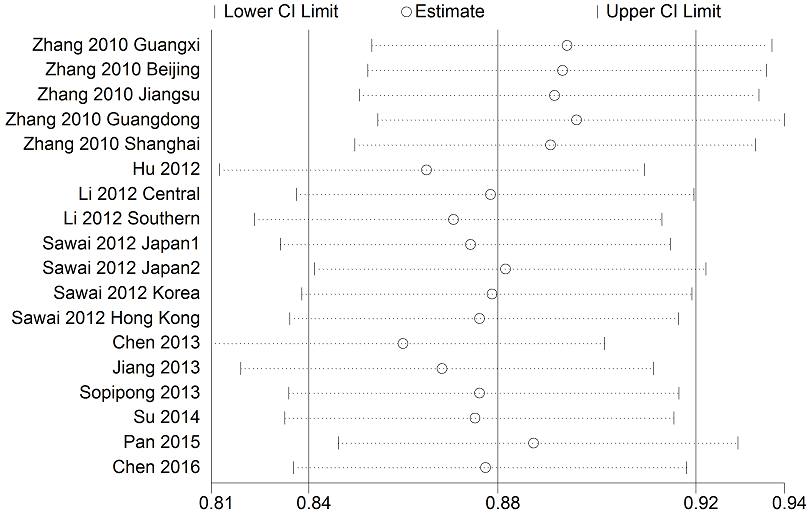


Figure S1 Sensitivity analysis of association between KIF1B polymorphism and HCC susceptibility under the allelic model G-allele vs A-allele
