# Supplementary material for: Association between KIF1B rs17401966 genetic polymorphism and hepatocellular carcinoma susceptibility: an updated meta-analysis
Source: BMC Med Genet. 2019 Apr 4;20:59. doi: 10.1186/s12881-019-0778-y (PMC6449895; doi:10.1186/s12881-019-0778-y)

Table S3 Sensitivity analysis of association between KIF1B polymorphism and HCC susceptibility under the co-dominant genotype model AG vs AA


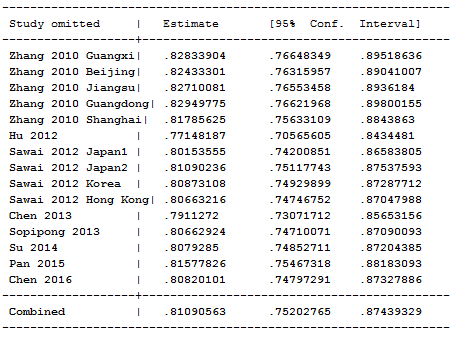

Supplement: Supplementary file 6 — Table S3. Sensitivity analysis of association between KIF1B polymorphism and HCC susceptibility under the co-dominant genotype model AG vs AA: The corresponding pooled OR were not changed when any single study was removed. (DOCX 81 kb) [file 12881_2019_778_MOESM6_ESM.docx]
